# A novel electrochemical immunosensor based on PdAgPt/MoS_2_ for the ultrasensitive detection of CA 242

**DOI:** 10.3389/fbioe.2022.986355

**Published:** 2022-08-24

**Authors:** Linlin Cao, Sumei Lu, Chengjie Guo, Wenqiang Chen, Yinan Gao, Diwen Ye, Zejun Guo, Wanshan Ma

**Affiliations:** ^1^ Department of Clinical Laboratory Medicine, The First Affiliated Hospital of Shandong First Medical University & Shandong Provincial Qianfoshan Hospital, Jinan, China; ^2^ Department of Clinical Laboratory, Zibo Central Hospital, Zibo, China

**Keywords:** label-free, electrochemical immunosensor, PdAgPt/MoS2, CA242, signal amplification

## Abstract

Dynamic monitoring of tumor markers is an important way to the diagnosis of malignant tumor, evaluate the therapeutic effect of tumor and analyze the prognosis of cancer patients. As a tumor marker of digestive tract, CA242 is often used to Assess the therapeutic effect of colorectal cancer and pancreatic cancer. In this study, immunosensor technology was used to detect CA242. PdAgPt nanocomposites, which have great advantages in biocompatibility, electrical conductivity and catalytic properties, were prepared by hydrothermal synthesis method. The prepared PdAgPt nanocomposites were loaded onto the surface of molybdenum disulfide (MoS_2_) with large surface area, and the new nanocomposites were synthesized. Using PdAgPt/MoS_2_ as signal amplification platform, the label-free CA242 electrochemical immunosensor has a wide detection range that extends from 1*10^−4^ U/ml to 1*10^2^ U/ml and a low detection limit (LOD, 3.43*10^−5^ U/ml) after optimization of experimental conditions. In addition, the CA242 immunosensor designed in this study also performed well in the evaluation of repeatability, selectivity and stability, and was successfully used for the detection of CA242 in human serum sample. Therefore, the label-free electrochemical immunosensor constructed in this study has a broad application prospect in the detection of clinical biomarkers.

## Introduction

Gastrointestinal neoplasms are one of the leading causes of cancer death around the world and seriously threaten human health (Arnold et al., 2020). Gastrointestinal tract tumors severely affect the survival of patients rate due to the characteristics of fast growth rate, early metastasis and occult onset (Hongmei Zeng et al., 2018; Howlader N et al., 2020). Despite the continuous improvement of surgical techniques and chemoradiotherapy schedules, improving early diagnosis and screening techniques is still the key step to elevated the prognosis of patients with gastrointestinal cancer (Sinicrope, 2022) As commonly used clinical examination indicators, tumor markers play very important roles in early screening, curative effect observation and prognosis evaluation of digestive tract tumors (Lech et al., 2016; Acharya et al., 2017). CA242 is a salivary acid sphingolipid antigen, and increased serum levels of CA242 has been used in the diagnosis of colorectal cancer, pancreatic cancer, gastric cancer and other cancers (Dou et al., 2019). Preoperative detection of CA242 combined with other tumor markers can improve prognostic prediction of patients undergoing colorectal cancer surgery (Kuang et al., 2020; Luo et al., 2020). However, there are few studies on CA242 detection methods (Tang et al., 2017; Du et al., 2019; Shen et al., 2022), so it is very important to develop sensitive and accurate detection methods for CA242.

Currently, the commonly used methods for detecting tumor markers include enzyme-linked immunosorbent assay (ELISA) (Dong et al., 2018), electrochemiluminescence (ECL) (Lu et al., 2021) and radioimmunoassay (RIA) (Elessawi et al., 2019). However, these methods need large operating equipment, cumbersome experimental operation and narrow linear range. Compared with these methods, electrochemical immunosensor has the advantages of high sensitivity, high speed and easy operation (Zhang et al., 2021). The electrochemical immunosensor (Aydin et al., 2021) is divided into sandwich electrochemical immunosensor and label-free electrochemical immunosensor. Label-free electrochemical immunosensor (Tan et al., 2020) has potential application value because of its high sensitivity, fast detection speed, low cost and no need for secondary antibody labeling.

In recent years, various nanomaterials, such as metal nanoparticles, graphene oxide and metal oxides, have been widely used to modify the surface of the induction electrode of immunosensors (Zhang et al., 2019). This nano-analog enzyme is superior to the natural horseradish peroxidase. For example, it is easy to prepare, low in cost, strong in stability (Tan et al., 2020). Tri-metal nanocomposites have attracted extensive attention due to their outstanding advantages in chemical stability, surface-to-volume ratio, electrical conductivity and biocompatibility. Compared with mono-metal or bimetallic nanomaterials, tri-metal nanocomposites have more catalytic active sites, which have attracted more and more attention in fermentation industry, food industry, biomedical detection, environmental monitoring and many other fields. Zhao et al. (2022) synthesized trimetal PdCuPt nanocomposites with excellent electrical conductivity and catalytic properties. The electrochemical signal amplification platform (PdCuPt/PPY/DCSC) was constructed by combining DCSC and PdCuPt. The prepared unlabeled electrochemical immunosensor has high sensitivity, wide dynamic range (50 fg/ml∼1 μg/ml) and low detection limit (16.03 fg/ml), realizing the ultra-sensitive detection of CTnI. Dong et al. (2020) prepared a novel sandwich signal amplification strategy based on Au/IRMOF-3 and using Pd@PtRh trimetallic nanomaterials as antibody labels. The feasibility of procalcitonin (PCT) electrochemical immunosensor was confirmed by chronoamperometry and differential pulse voltammetry (DPV), and the immunosensor has good linearity (20 fg/ml ∼ 100 ng/ml). Therefore, as excellent composite nanomaterials, trimetal nanocomposites have broad application prospects in the electrochemical field.

Palladium, silver and platinum nanocomposites, as trimetal nanocomposites, can efficiently catalyze H_2_O_2_, which are good candidates for the construction of immunosensors (Plauck et al., 2016; Li et al., 2020; Wu et al., 2021). However, metal materials tend to agglomerate due to their high specific surface area, which limit the extensive application (Bai et al., 2022; Li et al., 2022). Therefore, noble metals are able to function by uniformly loading them on the materials with large specific surface area, and have the advantage of cost-saving and improving the utilization rate (Yan et al., 2019; Tan et al., 2020). Currently, the common load materials include graphene oxide (Tan et al., 2020), molybdenum disulfide (Li et al., 2020), metal organic framework (Biswas et al., 2021), etc. MoS_2_, as a common two-dimensional material similar to graphene, is considered to be an excellent semiconductor material with good edge position, narrow band gap, large specific surface area, sensitivity to external environment, strong adsorption and other characteristics, and has been widely used in the preparation of immune sensors (Lu et al., 2020; Gong et al., 2022; Roy et al., 2022).

In this study, a novel label-free electrochemical immunosensor was constructed based on MoS_2_ supported PdAgPt nanocomposites to detect tumor marker CA242. Due to the specific binding of antigen and antibody, CA242 can be quantified according to the magnitude of the current signal. PdAgPt nanoparticles can accelerate electron transfer and effectively link antibodies through Pd-N and Pt-N bonds, which is an ideal matrix material for preparing immunosensors. In addition, the electrochemical immunosensor of peroxisase-like activity constructed by PdAgPt nanoparticles loaded with molybdenum disulfide can co-amplify the current signal. The PdAgPt/MoS_2_ nanocomposites have good dispersibility, conductivity and biocompatibility. Therefore, the fabricated CA242 immunosensor has high sensitivity, stability and selectivity, and provide a new detection method for the diagnosis and treatment of digestive tract tumors.

## Materials and methods

### Reagents and equipments

Na_2_PdCl_4_ and AgNO_3_ were purchased from Shanghai Maclin Biochemical Technology Co., LTD. H_2_PtCl_6_ was achieved from Sigma Aldrich Trading Co., LTD. Cetyltrimethylammonium chloride (CTAC), L-cysteine, Aminopropyl triethoxysilane (APTES) and Ascorbic acid (AA) were bought from Shanghai Maclin Biochemical Technology Co., LTD. Na_2_MoO_4_.2H_2_O was purchased from Shanghai Jiu ding Chemical Technology Co., LTD. Bovine serum albumin (BSA), CA242, and CA242 antibody were purchased from Shanghai Lingchao Biotechnology Co., LTD. Phosphate buffer solutions (PBS) were prepared with Na_2_HPO_4_ and KH_2_PO_4_. Serum samples were collected from Zibo Central Hospital. The ultra-pure water (18.25 Ω) used was self-made by the laboratory. Hydrogen peroxide (H_2_O_2_, 30 wt%) was obtained from Yantai Shuangshuang Chemical Co., Ltd.

Electrochemical measurement was performed using electrochemical workstation CHI660E. Tecnai G2 F20 transmission electron microscope was used to collect the images and analyze the morphologic characteristics and composition of the sample.

### Preparation of PdAgPt nanoparticles

First, 1.5 ml Na_2_PdCl_4_ (5 mM), 1.5 ml AgNO_3_ (5 mM) and 1.5 ml H_2_PtCl_4_ (5 mM) solution were added successively to 15 ml CTAC (100 mM) solution, and were fully mixed. Then quickly added 150 μl newly-prepared AA solution (100 mM) to the mixture, poured the mixture into 50 ml POLY tetrafluoron liner (PTFE), assembled the reactor steel sleeve, heat in an oven at 95°C for 2 h, and centrifuged for the collection of products.

### Preparation of molybdenum disulfide

Na_2_MoO_4_.2H_2_O (0.125 g) and secondary deionized water (25.0 ml) were added to a beaker, mixed well, and dissolved with a glass stick. After the solution was fully dissolved, the pH value of the solution was measured with a pH meter, and the pH value was adjusted to 6.5 by adding hydrochloric acid (0.1 M). Then L-cysteine (0.25 g) and the prepared solution were mixed under ultrasonic shock for 10 min. The solution was then added to a 50 ml polytetrafluoroethylene (PTFE) liner, the reactor steel jacket was assembled and heated in an oven (200°C, 16 h). Finally, the collected product was washed with secondary deionized water for 3 times and dried for later use.

### Synthesis of functionalized PdAgPt nanoparticles supported by MoS_2_

To synthesize PdAgPt/MoS_2_, amino functionalized molybdenum disulfide (NH_2_-MoS_2_) was prepared. First, 4.0 mg molybdenum disulphide and 5.0 ml anhydrous ethanol were added into a round-bottom flask and treated with ultrasonic shock for 15 min. Then, 50 μl 3-aminopropyltriethoxysilane (APTES) was added to the flask under magnetic agitation and heated at 100°C for 2 h at reflux. After the solution cooled naturally, the product was washed with deionized water twice and centrifuged for use.

Under ultrasonic condition, 2.0 ml PdAgPt and 2.0 ml NH_2_-MoS_2_ were added into the test tube and mixed for 30 min. Then shake well in oscillator (4°C) for 8 h. Finally, the product PdAgPt/MoS_2_ was collected by centrifugation.

### Construction of CA242 electrochemical immunosensor

**FIGURE 1:**
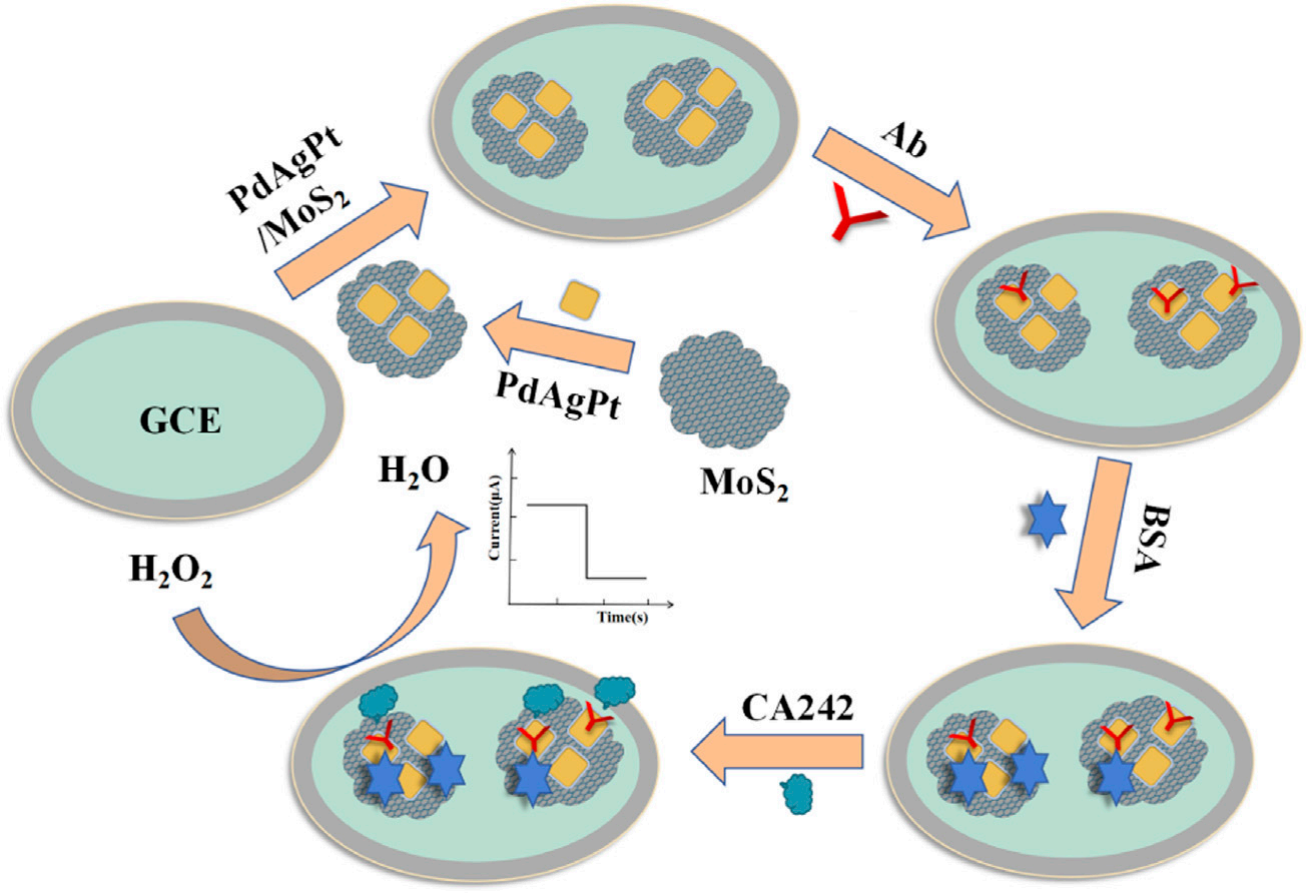
The fabrication process of CA242 immunosensor.

As shown in Figure 1, the CA242 label-free sensor was successfully constructed on the bare glassy carbon electrode (GCE). Firstly, the surface of GCE was modified with 6 ul PdAgPt/MoS_2_ (2 mg/ml) and dried in air to prepare the working electrode. Then, 6 uL anti-CA242 (1 U/ml) was modified on the electrode as an antigen collector of CA242. 3uL 1 wt% bovine serum albumin (BSA) was added continuously on the surface of the working electrode to seal the non-specific active sites. After the sensor was constructed, the reaction conditions were optimized, and then CA242 with different concentration gradients was added to the working electrode to draw the working curve. Finally, the CA242 electrochemical immunosensor was successfully assembled and stored in a 4°C refrigerator.

### Electrochemical measurement

Electrochemical measurement was carried out with three electrode system of electrochemical workstation. The current-time (i-t) curve is recorded by amperometric technique. The electrolyte was phosphate buffer solution (PBS, 10 ml, pH = 7.17), the initial potential was set at 0.25 V, and the catalytic activity substrate was H_2_O_2_ (10 μl, 5.0 mol/L). Electrochemical impedance spectroscopy (EIS) was used to characterize the modification process of the sensor. The electrolyte was prepared by 0.1 mol/L KCl, 2.5 mmol/L [Fe(CN)_6_]^4-^ and 2.5 mmol/L [Fe(CN)_6_]^3-^. The open-circuit voltage was set to 0.20 V, and the frequency range is 0.1 Hz–100 kHz. The electrolyte used for cyclic voltammetry (CV) measurements was prepared from 5.0 mmol/L [Fe(CN)_6_]^3-^ and 0.1 mol/L KNO_3_ at a scanning rate of 0.10 V/s.

## Results and Discussion

### Characterization of PdAgPt/MoS_2_

**FIGURE 2:**
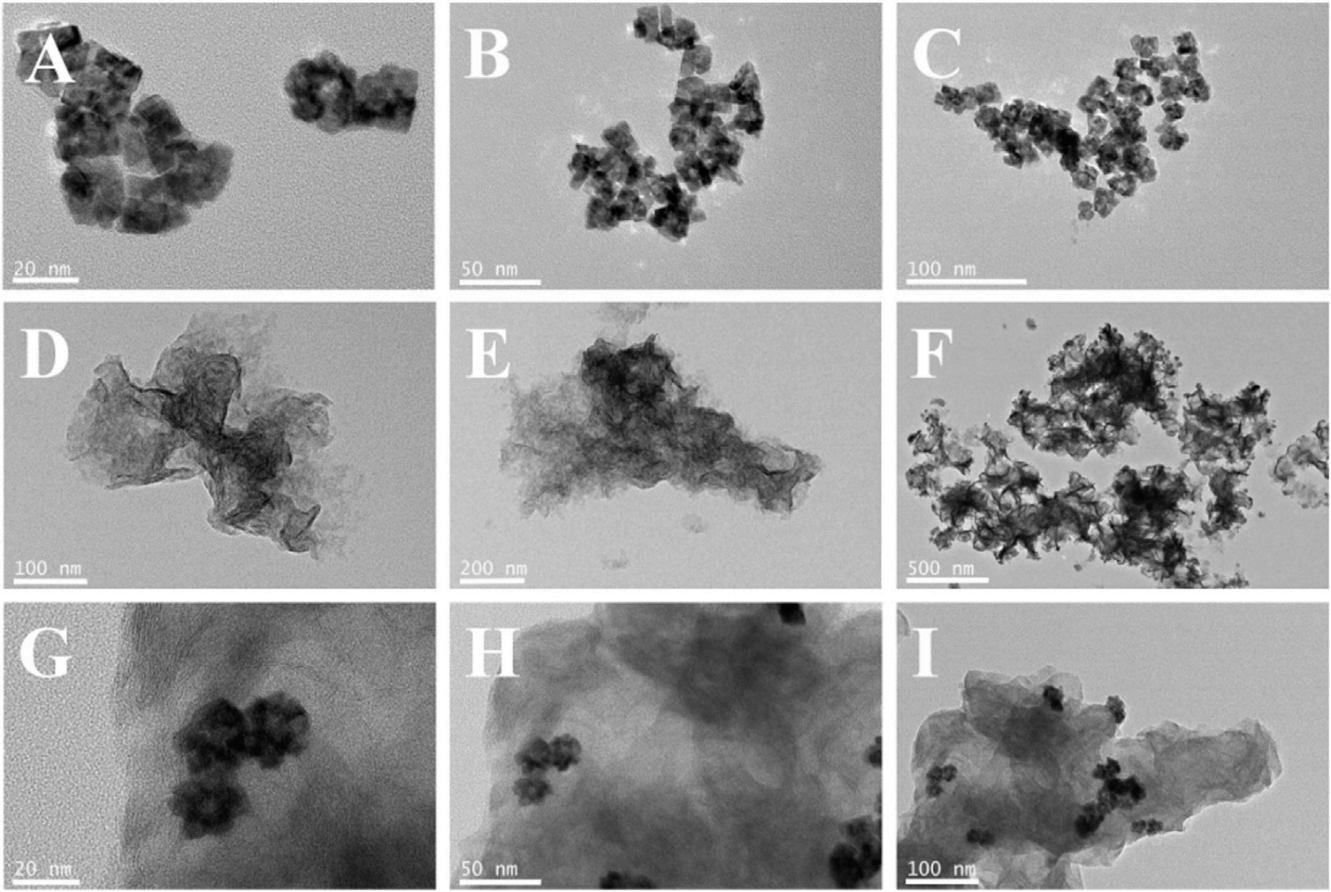
**(A–C)** TEM images of PdAgPt nanocomposites. **(D–F)** TEM images of MoS2. **(G–I)** TEM images of PdAgPt/MoS_2_ nanocomposites.

**FIGURE 3:**
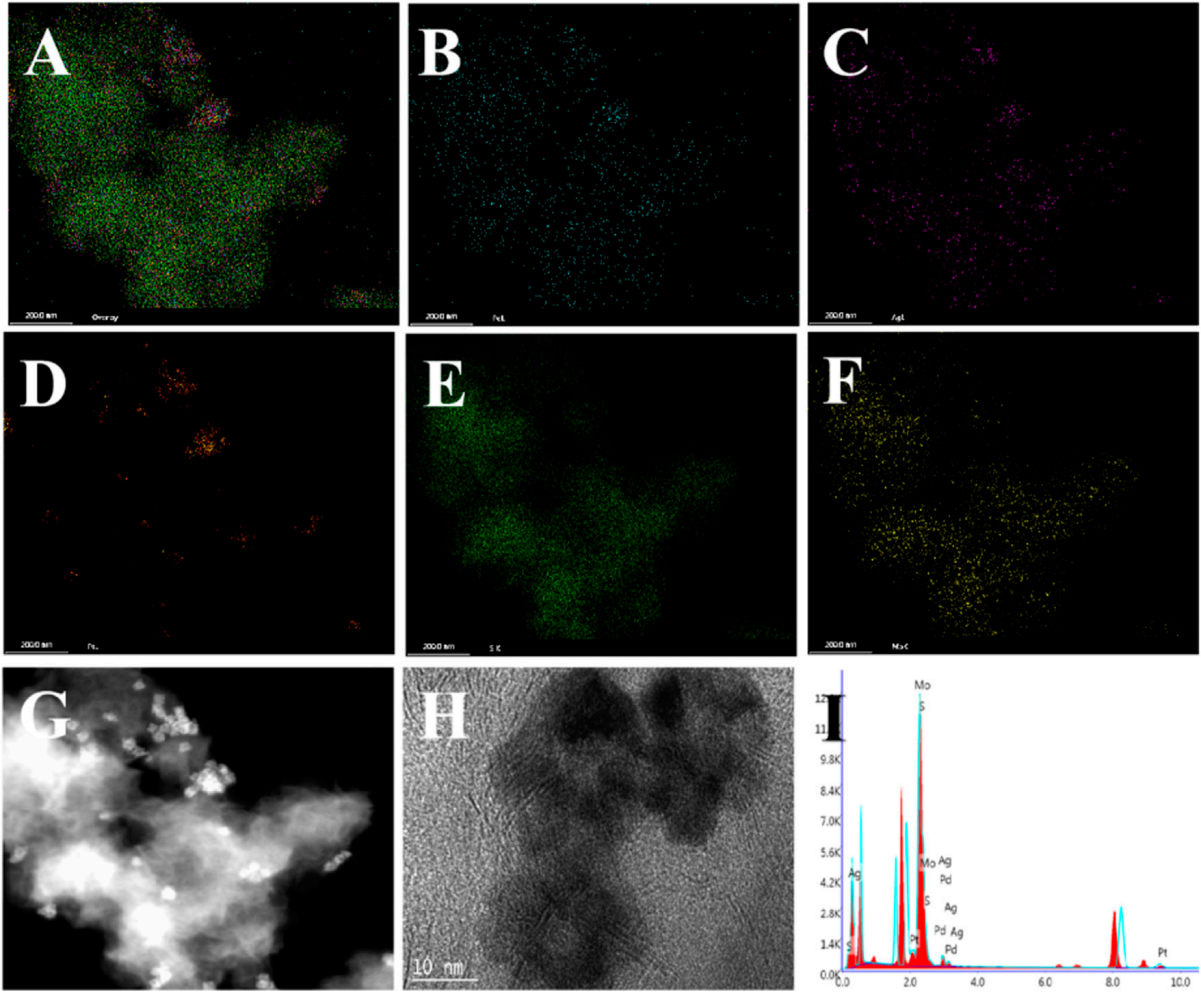
Characterization of PdAgPt/MoS_2_ nanocomposites. **(A–F)** Elemental mappings of **(A)** PdAgPt/MoS_2_ nanocomposites, **(B)** Pd, **(C)** Ag, **(D)** Pt, **(E)** S, and **(F)** Mo.**(G–H)** TEM image of PdAgPt/MoS_2_ nanocomposites. **(I)** X-ray energy (EDX) spectrum of PdAgPt/MoS_2_ nanocomposites.

Figures 2A–C; Supplementary Figure S1 clearly show the flower-like PdAgPt NPs with the diameter of approximately 20 nm and uniform distribution. Aminated molybdenum disulfide is a folded paper structure with a large surface area (Figures 2D–F). Transmission electron microscope (TEM) images of PdAgPt/MoS_2_ show that PdAgPt NPs are successfully and uniformly connected to aminated molybdenum disulfide (Figures 2G–I). Both SEM elemental mapping images and energy dispersive X-ray (EDX) analysis of PdAgPt/MoS_2_ showed that the nanocomposites were composed of Pd, Ag, Pt, Mo and S elements (Figure 3), indicating a successful combination of PdAgPt NPs and MoS_2_. In summary, NH_2_-MoS_2_ loaded PdAgPt NPs with large surface and abundant active sites were successfully prepared.

### Electrochemical properties of PdAgPt/MoS_2_

The sensitivity of the electrochemical immunosensor is affected by the catalytic activity of the electrode surface, so the catalytic performance of nanomaterials is a key factor affecting the performance of the immunosensor. According to the references (Van Venrooij and Koper, 1995; Felix and Angnes, 2018; Wu et al., 2019), Amperometric i-t technique is used to detect antigen content through different current signals generated on the electrode surface. The reduction reaction of hydrogen peroxide occurs under the catalysis of the substrate material, and the current generated on the electrode surface is proportional to the concentration of antigen. The mechanism of hydrogen peroxide catalyzed by substrate materials is as follow:

H_2_O_2_ + 2H^+^ + 2e^−^ → 2H_2_O_2_

**FIGURE 4:**
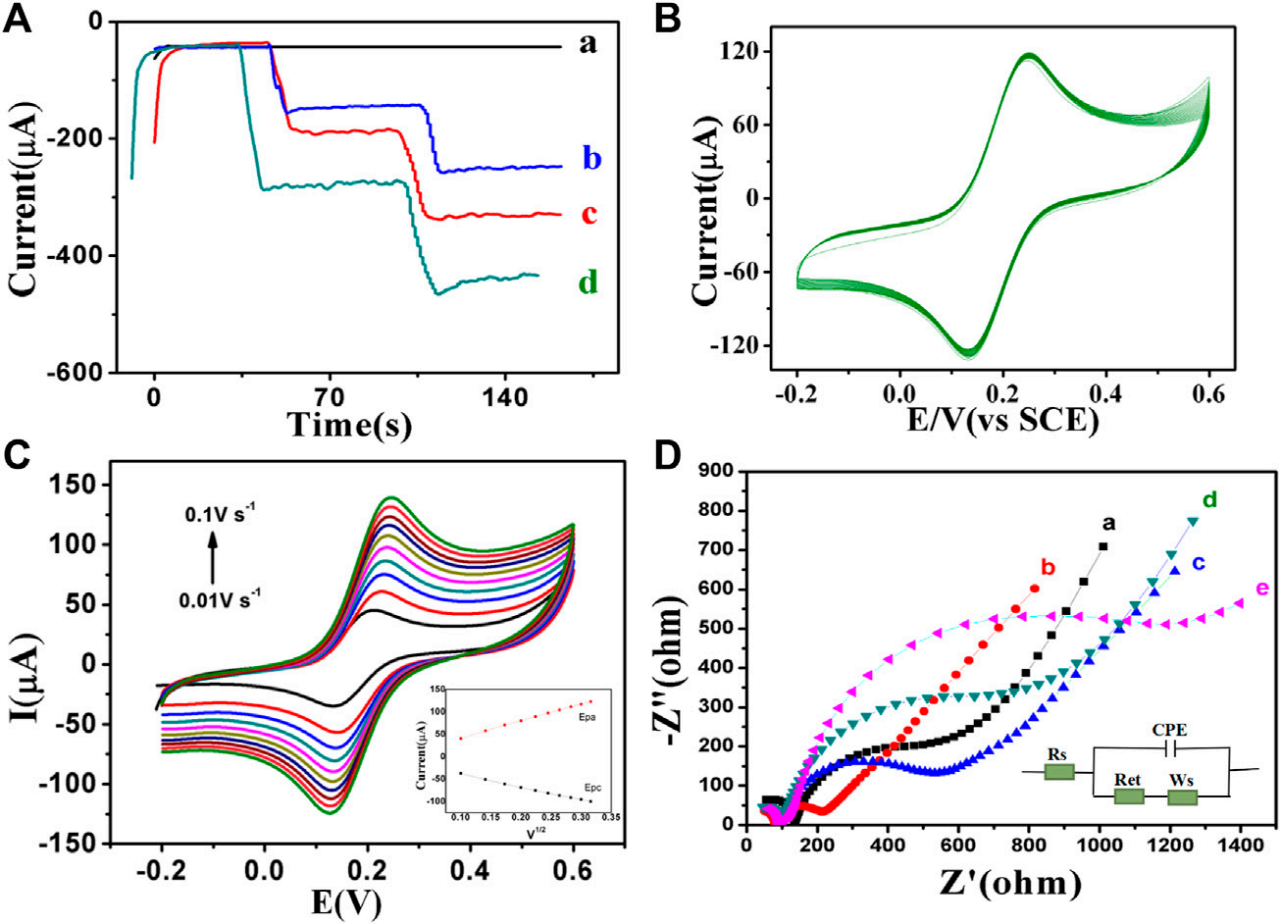
Electrochemical characterization of PdAgPt/MoS_2_ nanocomposites. **(A)** The amperometric current–time experimental curves: GCE (a), PdAgPt (b), MoS_2_(c), PdAgPt/MoS_2_ (d). **(B)** Cyclic voltammetry (CV) diagram of PdAgPt/MoS_2_ modified GCE continuously sweeping for 40 times at the scan rate of 0.1 V/s. **(C)** The CV curves of PdAgPt/MoS_2_ modified electrodes at different scan rates (0.01 V/s–0.1 V/s); Inset: the linear relationship of Epa (the red line, ipa = 380.34v^1/2^ + 3.5149) and Epc (the black line, ipc = −285.43v^1/2^–11.069) between the anodic and cathodic peak currents versus scan rate of PdAgPt/MoS_2_. **(D)** EIS of GCE (a), PdAgPt/MoS_2_/GCE (b), anti-CA242/PdAgPt/MoS_2_/GCE (c), BSA/anti-CA242/PdAgPt/MoS_2_/GCE (d), and CA242/BSA/anti-CA242/PdAgPt/MoS_2_/GCE (e).

In this paper, amperometric i-t method was used to study the response current of H_2_O_2_ reduction catalyzed by GCE, PdAgPt, MoS_2_ and PdAgPt/MoS_2_ (Figure 4A). After the background current was stabilized, H_2_O_2_ was injected into PBS at pH = 6.98, and the modified material showed a typical ampere i-t curve. As can be seen from the figure, the catalytic capacity of PdAgPt and MoS_2_ on H_2_O_2_ is slightly lower (Figure 4A curve b-c), but when PdAgPt is loaded on MoS_2_, the current signal of PdAgPt/MoS_2_ is stronger (Figure 4A curve d), indicating the catalytic performance is enhanced. The synergistic effect of PdAgPt and MoS_2_ can effectively promote the decomposition of H_2_O_2_.

The stability of substrate material is also very important for the performance evaluation of immunosensor. As shown in Figure 4B, GCE was modified by PdAgPt/MoS_2_ continuously sweeping for 20 times at the scan rate of 0.1 V/s using cyclic voltammetry to generate corresponding curves. The peak current and peak potential did not change significantly, indicating that PdAgPt/MoS_2_ nanocomposites have good stability. Therefore, PdAgPt/MoS_2_ nanocomposites are good substrate materials for the construction of immune sensor.

In addition, the electroactive surface area of PdAgPt/MoS_2_ can be obtained by calculating CV values. At the scanning rate of 0.01–0.10 V/s, the peak current of both cathode and anode increases gradually with the increase of scanning rate (Figure 4C). Oxidation peak potential is positive, reduction peak potential is negative. A good linear relationship between the peak anodic (or cathodic) current (ip) and the square root of the scanning rate (V^1/2^) can be seen in the Figure 4C, and the redox reaction on the surface of GCE electrode is proved to be a diffusion-controlled process. According to Randles-Sevcik formula (ip = 2.686 × 10^5^N^3/2^AD^1/2^CV^1/2^), the electroactive surface area of PdAgPt/MoS_2_ is 0.1143 cm2. This indicates that PdAgPt/MoS_2_ has better electroactive area and can provide more catalytic active sites, that is, the reduction reaction performance of substrate H_2_O_2_ is better (see Supplementary Material S1).

The electrochemical characteristics of biosensor interface were detected by electrochemical impedance method (EIS). As shown in Figure 4D, when PdAgPt/MoS_2_ nanomaterial is modified to bare GCE, Ret (curve A) of PdAgPt/MoS_2_ is significantly lower than that of bare GCE (curve b), indicating that PdAgPt/MoS_2_ has good conductivity and can accelerate electron transfer. Then we modified anti-CA242 (curve c), BSA (curve d) and CA242 (curve e) on the surface of PdAgPt/MoS_2_/GCE, and the corresponding resistance values increased successively (Supplementary Table S1). This is because anti-CA242, BSA and CA242, as macromolecular proteins, all block electron transfer. The resistance variation trend proves that CA242 electrochemical immunosensor was successfully constructed.

### Optimization of experimental conditions for immunosensor

**FIGURE 5:**
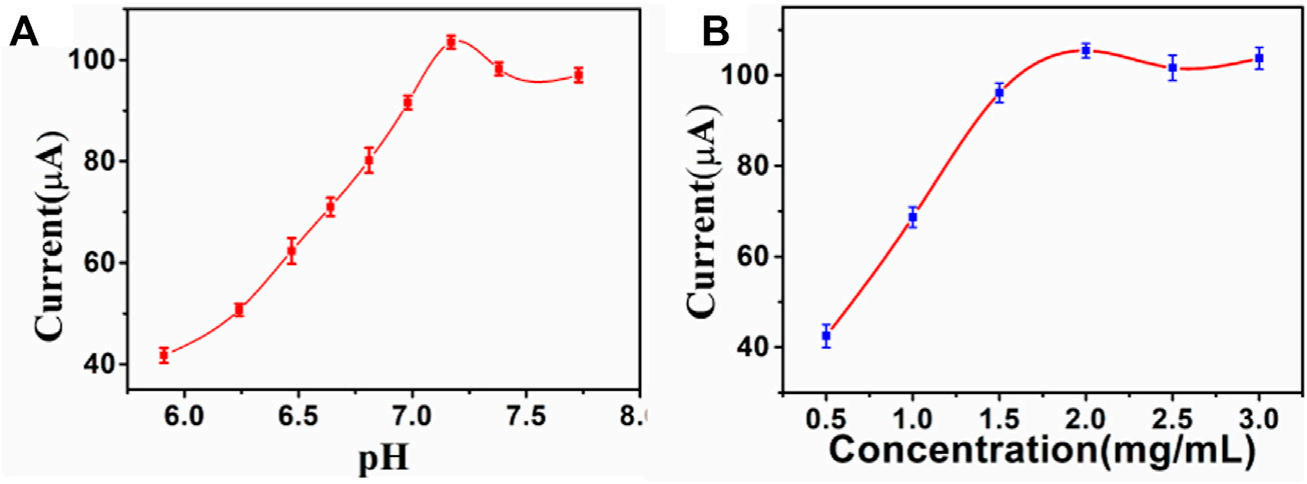
Optimize experimental conditions: **(A)** pH of PBS, **(B)** Concentration of PdAgPt/MoS_2_, error bar = RSD (*n* = 4).

After the CA242 electrochemical immunosensor was constructed, optimal experimental conditions such as substrate material concentration and pH were optimized to obtain more accurate tumor marker measurements. As shown in Figure 5A, the pH used in this study are 5.91, 6.24, 6.47, 6.64, 6.81, 6.98, 7.17, 7.38, and 7.71 respectively. The experimental results show that different pH values have different current response. pH of PBS from 7.17 to 7.71, the current signal continuously decreased. The current signal reaches the peak value at pH 7.17, so the optimal pH value of the sensor is 7.17. PdAgPt/MoS_2_ concentration from 0.5 mg/ml to 2 mg/ml, the current signal reaches the peak, so the optimal concentration of PdAgPt/MoS_2_ is 2 mg/ml (Figure 5B).

### Analytical performance of CA242 immunosensor

**FIGURE 6:**
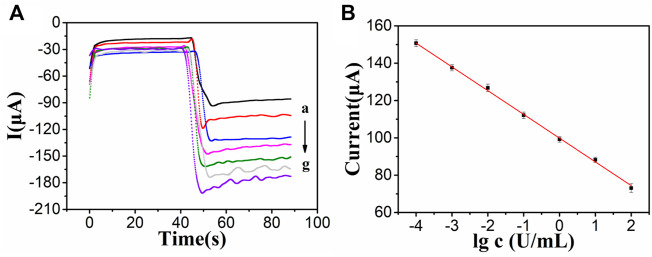
**(A)** i-t measurements of different concentrations of CA242: 100 U/ml(a), 10 U/ml (b), 1 U/ml (c), 0.1 U/ml (d), 0.01 U/ml (e), 0.001 U/ml (f), 0.0001 U/ml (g). **(B)** The linear relationship between the logarithm of CA242 concentration and the current signal, error bar = SD (*n* = 4).

After the optimization of experimental conditions, then we used PdAgPt/MoS_2_ as the signal amplification platform to detect current signals by amperometric method and generate i-t curves. Adding tumor marker CA242 with different concentrations will generate different current signals. As shown in Figure 6A, with the increase of the concentration of CA242, the response of the current signal decreases continuously, because the electron transfer process is obstructed with the increase of the concentration of antigen. The fitting operating curve in Figure 6B shows a linear relationship between the current response and the logarithm of CA242 concentration in the range of 0.0001 U/ml to 100 U/ml. The regression equation is the fitting operating curve y = -12.72lgC+99.86, and the correlation coefficient is 0.9985. The detection limit of CA242 was 3.43*10^−5^ U/ml (S/N = 3). At the same time, the constructed CA242 immunosensor was compared with the CA242 immunosensor reported in literature. It can be seen from Supplementary Table S2 that the linear range and minimum detection limit of the immune sensor constructed in this study are both good, because PdAgPt/MoS_2_ nanocomposite material has excellent electrical conductivity and strong catalysis to substrate H_2_O_2_.

### Reproducibility, stability and sensitivity

**FIGURE 7:**
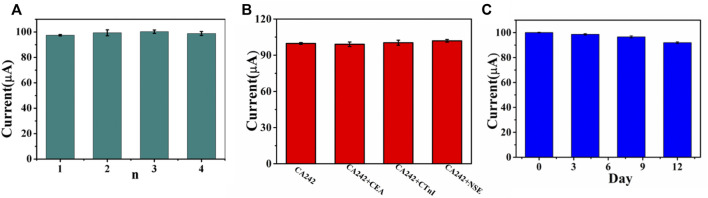
**(A)** Amperometric change response of biosensor to four different electrodes treated in same way. **(B)** Current responses of the immunosensor to 1U/ml CA242, 1U/ml CA242+1 ng/ml CEA, 1U/ml CA242+1 ng/ml CTnI, 1U/ml CA242+1 ng/ml NSE, error bar = RSD (*n* = 4). **(C)** The stability study of the CA242 immunosensor, error bar = RSD (*n* = 3).

Repeatability, specificity and stability are three important indexes for the performance evaluation of immunosensor. Under the same conditions, four CA242 immunosensors were prepared. The relative standard deviation (RSD) of electrode measurement for CA242 (1 U/ml) was 1.19%, indicating that the immunosensor had good repeatability (Figure 7A). In order to study the specificity of the immunosensor, CEA (1 ng/ml), CTnI (1 ng/ml) and NSE (1 ng/ml) were added to CA242 (1 U/ml) solution for detection. As shown in Figure 7B, RSD is 1.22%, which means that the selectivity of the immunosensor is acceptable. Then, the immunosensor was placed in the refrigerator of 4°C and the current response test was conducted every 4 days. Compared with the initial data, the current response value of the sensor decreased by 8.09% (Figure 7C), indicating that the immunosensor had good stability.

### Real sample analysis

In order to investigate the accuracy and operability of the immunosensor, human serum CA242 was determined by standard recovery test. As shown in Supplementary Table S3, CA242 standard solutions at 1.0 U/ml, 5.0 U/ml, and 10.0 U/ml were added into serum samples, and the calculated recoveries were 98.0%, 104% and 107%. The relative standard deviations (RSDs) were 4.53%, 3.22%, and 5.48%, respectively. Therefore, the immunosensor constructed in this study can detect tumor marker CA242 sensitively and has great application potential.

## Conclusions

In this study, an unlabeled electrochemical immunosensor based on PdAgPt/MoS_2_ was constructed for ultra-sensitive quantitative detection of tumor marker CA242. PdAgPt/MoS_2_ nanocomposites as signal amplification platform can effectively capture the antigen to be measured, with a wide detection range (1*10^−3^ U/ml–1*10^2^ U/ml) and ultra-low detection limit (3.43*10^−5^U/ml). The sensor showed good reproducibility, selectivity and stability, and was tested in actual samples, which can be used for dynamic monitoring of tumor markers. Therefore, this immunosensor may be developed into a good method for clinical detection in the future, and provide effective guidance for the detection of other clinical diagnostic markers.

## Data Availability

The original contributions presented in the study are included in the article/Supplementary Material, further inquiries can be directed to the corresponding author.
