# Supplementary material for: A novel electrochemical immunosensor based on PdAgPt/MoS2 for the ultrasensitive detection of CA 242
Source: Front Bioeng Biotechnol. 2022 Aug 24;10:986355. doi: 10.3389/fbioe.2022.986355 (PMC9449583; doi:10.3389/fbioe.2022.986355)
Supplement: Supplementary file 1 [file DataSheet1.docx]

Supplementary Material

A Novel Electrochemical Immunosensor based on PdAgPt/MoS_2_ for the Ultrasensitive Detection of CA 242

Linlin Cao^1, 2#^, Sumei Lu^1#^, Chengjie Guo^2^, Wenqiang Chen^2^, Yinan Gao^1^, Diwen Ye^1^, Zejun Guo^1^, Wanshan Ma^1*^

1. ***Department of Clinical Laboratory Medicine, The First Affiliated Hospital of Shandong First Medical University & Shandong Provincial Qianfoshan Hospital, Shandong Medicine and Health Key Laboratory of Laboratory Medicine. Jinan, China***
2. ***Department of Clinical Laboratory, Zibo Central Hospital, Zibo, China***

*** Correspondence:**Wanshan Ma
[mwsqianyi@163.com](mailto:mwsqianyi@163.com)

^#^ Linlin Cao and Sumei Lu contributed equally to this work.

**Figure S1** TEM of PdAgPt/MoS_2_.


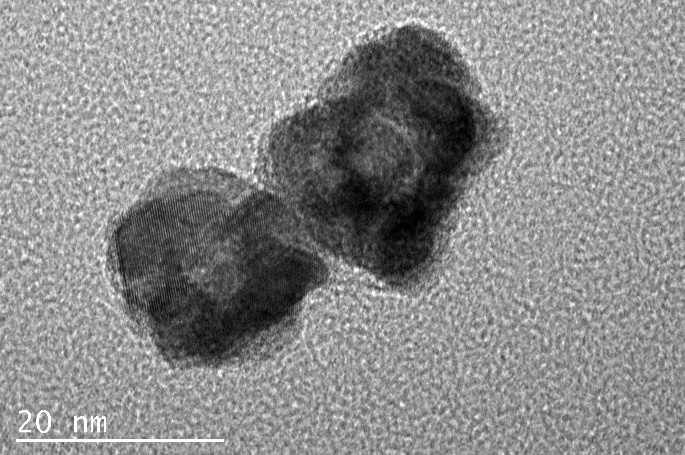


**Electroactive area**

The electroactive area of PdAgPt/MoS_2_ modified GCE was calculated by Randles-Sevcik equation. The calculation formula is

$$i_{P} = 2.686 \times{10}^{5} D^{\frac{1}{2}} n^{\frac{3}{2}}A C v^{\frac{1}{2}}$$

Among them, D was the diffusion coefficient, n was the number of electron transfers, A was the electroactive area，C was the concentration of [Fe(CN)^6^]^3-^, v was the sweep speed, and ip was the peak current. According to the equation, the electroactive area（A） of PdAgPt/MoS_2_ modified GCE was 0.1143 cm^2^.

**Table S1** The Ret of EIS

| Electrode | Ret (Ω) |
| --- | --- |
| GCE (a) | 353.3 |
| PdAgPt/MoS2/GCE (b) | 142.3 |
| Anti-CA242/ PdAgPt/MoS2/GCE (c) | 303.9 |
| BSA/Anti-CA242/ PdAgPt/MoS2/GCE (d) | 475.2 |
| CA242/BSA/Anti-CA242/ PdAgPt/MoS2/GCE (e) | 837.6 |

**Table S2 Comparison of different detection methods for CA242**

| Signal amplification platform | Methods | Linear range | LOD | References |
| --- | --- | --- | --- | --- |
| rGO-Au-Pd | Electrochemical Immunosensor | 10^-3^ U/mL ~ 10^4^ U/mL | 1.54 mU/mL | (Du et al., 2019) |
| SnS_2_/Cd-ZnIn_2.2_S_y_ | Photoelectrochemical Immunosensor | 10^-4^U/mL ~ 10^2^ U/mL | 0.306 mU/mL | (Li et al., 2022) |
| g-C_3_N_4_@PtNPs and luminol-AgNPs@ZIF-67 | Electrochemiluminescence Immunosensor | 0.0005 ~ 10 U/mL | 0.16 mU/mL | (Mo et al., 2021) |
| Zn–CP, CHIT–AuNPs | Electrochemical Immunosensor | 1 ~ 150 U/mL | 0.4 U/mL | (Rong et al., 2016) |
| GOx-ZIF-8/Au-rGO | Amperometric Immunosensor | 10^-3^ ~ 10^3^ U/mL | 0.69 mU/mL | (Zheng and Ma, 2019) |
| CuPDA, PEI-GO | Electrochemical Immunosensor | 10^-4^ ~ 10^2^ U/mL | 0.21 mU/mL | (Zheng et al., 2018) |
| SnS_2_QDs@MIL-101 | Electrochemiluminescence Immunosensor | 0.1mU/mL ∼10^2^ U/mL | 0.15 mU/mL | (Shen et al., 2022) |
| SA-Pb^2+^-GO | [Electrochemical Immunoassay](https://www.sciencedirect.com/topics/chemistry/electrochemical-immunoassay) | 0.005 U/mL∼ 500 U/mL | 0.67 mU/mL. | (Tang et al., 2017) |
| **PdAgPt/MoS_2_** | [**Electrochemical Immunoassay**](https://www.sciencedirect.com/topics/chemistry/electrochemical-immunoassay) | **10^-4^ ∼10^2^ U/mL** | **0.34 mU/mL** | **This work** |

**Table S3** Detection of the CA242 in human serum samples.

| Sample | Concentration of CA242 (U/mL) | The addition (U/mL) | The detection (U/mL) | RSD (%, **n** = 3) | Recovery (%) |
| --- | --- | --- | --- | --- | --- |
| 1 | 10.6 | 1.0 | 11.0,11.6,12.1 | 4.53 | 98.0 |
| 2 | 10.6 | 5.0 | 16.4,15.5,15.5 | 3.22 | 105 |
| 3 | 10.6 | 10.0 | 22.3,21.5,20.0 | 5.48 | 107 |

**Reference**

Du, X., Zheng, X., Zhang, Z., Wu, X., Sun, L., Zhou, J., et al. (2019). A Label-Free Electrochemical Immunosensor for Detection of the Tumor Marker CA242 Based on Reduced Graphene Oxide-Gold-Palladium Nanocomposite. *Nanomaterials (Basel)* 9(9). doi: 10.3390/nano9091335.

Li, Y., Cao, L., Shen, C., Meng, F.-N., Li, Y., Wang, S., et al. (2022). Heterostructure photoelectrochemical immunosensor based on flower-like refraction structure Cd-ZnIn2.2Sy sensitized 2D hexagonal SnS2 nanoplates for CA242 detection. *Sensors and Actuators B: Chemical* 367. doi: 10.1016/j.snb.2022.132186.

Mo, G., He, X., Qin, D., Meng, S., Wu, Y., and Deng, B. (2021). Spatially-resolved dual-potential sandwich electrochemiluminescence immunosensor for the simultaneous determination of carbohydrate antigen 19-9 and carbohydrate antigen 24-2. *Biosens Bioelectron* 178**,** 113024. doi: 10.1016/j.bios.2021.113024.

Rong, Q., Feng, F., and Ma, Z. (2016). Metal ions doped chitosan–poly(acrylic acid) nanospheres: Synthesis and their application in simultaneously electrochemical detection of four markers of pancreatic cancer. *Biosensors and Bioelectronics* 75**,** 148-154. doi: 10.1016/j.bios.2015.08.041.

Shen, C., Li, Y., Li, Y., Wang, S., Li, Y., Tang, F., et al. (2022). A double reaction system induced electrochemiluminescence enhancement based on SnS2 QDs@MIL-101 for ultrasensitive detection of CA242. *Talanta* 247**,** 123575. doi: 10.1016/j.talanta.2022.123575.

Tang, Z., Fu, Y., and Ma, Z. (2017). Multiple signal amplification strategies for ultrasensitive label-free electrochemical immunoassay for carbohydrate antigen 24-2 based on redox hydrogel. *Biosens Bioelectron* 91**,** 299-305. doi: 10.1016/j.bios.2016.12.049.

Zheng, Y., and Ma, Z. (2019). Multifunctionalized ZIFs nanoprobe-initiated tandem reaction for signal amplified electrochemical immunoassay of carbohydrate antigen 24-2. *Biosens Bioelectron* 129**,** 42-49. doi: 10.1016/j.bios.2019.01.016.

Zheng, Y., Zhao, L., and Ma, Z. (2018). pH responsive label-assisted click chemistry triggered sensitivity amplification for ultrasensitive electrochemical detection of carbohydrate antigen 24-2. *Biosens Bioelectron* 115**,** 30-36. doi: 10.1016/j.bios.2018.05.026.
